# A comprehensive review on sustainable coastal zone management in Bangladesh: Present status and the way forward

**DOI:** 10.1016/j.heliyon.2023.e18190

**Published:** 2023-07-22

**Authors:** Mosa. Tania Alim Shampa, Nusrat Jahan Shimu, K M Azam Chowdhury, Md. Monirul Islam, Md. Kawser Ahmed

**Affiliations:** a Department of Oceanography, University of Dhaka, Dhaka-1000, Bangladesh; b Department of Fisheries, University of Dhaka, Dhaka-1000, Bangladesh

**Keywords:** Sustainable management, Coastal zone, Coastal resources, Coastal hazards, Environmental impact, Coastal development, Tropical cyclones, Sea level rise, Mangrove forests

## Abstract

Bangladesh, a coastal developing nation with a diverse sustainable biodiversity of natural resources is currently focused upon by international communities as a result of its high potential of the coastal zone (CZ) with natural gas. Sustainable Coastal Zone Management (SCZM) is key to its national development. SCZM refers to the management of coastal resources in order to provide secure and alternative livelihoods, as well as to manage all types of coastal hazards and social and cultural well-being in order to ensure long-term productivity and minimize environmental impact. This paper aims to delineate the current initiatives and status of coastal management in Bangladesh, highlighting key issues such as climate changes, sea level rise, tropical cyclones, coastal and marine pollution, coastal erosions, saltwater intrusions, and mangrove degradations as well as the future trend in Bangladesh which will facilitate sustainable development by emphasizing the social, ecological, and economic pillars of sustainability. Unsustainable coastal development practices in Bangladesh are going to damage the coastal ecosystems, particularly mangrove forests and coral reefs, which provide protection against tropical cyclones caused by global climate change and coastal erosions. The paper concludes by outlining a roadmap toward achieving SCZM in Bangladesh. The road to achieving SCZM requires collaboration, integration of scientific research, policy frameworks, community engagement, capacity building, and long-term commitment from all stakeholders involved. So, it is required to address all kinds of coastal issues and reframes all existing coastal management practices to ensure a healthy productive ecosystem to achieve SCZM as well as the sustainable development of the country.

## 1 Introduction

Coastal zones (CZs) are defined as transition zones where land meets the sea. It's among the most densely populated areas of the globe and are the sites of intense economic activity in the developed nations [1]. It is dynamic, diverse, and has no specific natural boundaries. CZs are distinctive due to particular characteristics including tides, coral reefs, barrier islands, sea shores, and storm waves [2]. The CZ of Bangladesh is about 47,203 km^2^, consisting of 19 coastal districts and a 710 km-long coastline, or 200 nautical miles of Exclusive Economic Zone (EEZ). Around 35.1 million people live here [3], with a population density of 743 people per square kilometer [4]. The CZ of Bangladesh is blessed with the largest complex estuarine systems, mangroves, coral reefs, deltas, wetlands, saltmarshes, seaweeds, coastal and marine fisheries, floras, faunas, sea salts, beach minerals, sand dunes, and other resources [5]. However, the coastal region of Bangladesh is now facing natural and anthropogenic pressures and threats. Both the growth of the local population and the level of economic pressure in the CZ will continue to rise not just in the not-too-distant future, but also in the centuries to come [6]. The unique geographic location of Bangladesh makes it one of the most vulnerable countries in the world due to climate change and several extreme events every year like tropical cyclones, tidal surges, storm surges, floods, sea level rises, saltwater intrusions, harmful wave actions, erosions, etc. [7]. According to the Bangladesh Bureau of Statistics (2022) [8], over 43.8 million people live in the coastal regions of Bangladesh. These people face the risks of frequent natural disasters, the effects of climate change, and restricted socio-economic growth [9] [3]. Understanding the long-term and large-scale evolution of the CZ is essential for sustainable coastal management and the related evaluations of coastal effects. This is especially true in light of the predicted global climate change [10]. It is now urgently needed to manage our coastal regions and conserve our coastal and marine living and non-living resources. To achieve this, there need for SCZM. SCZM means managing the coastal resources, environment, and economy in a way that will benefit the current generation as well as future generations. The rapid growth of socioeconomic activity in coastal and estuary areas during the past few decades rendered management efforts much more difficult. The implementation of sustainable development patterns in economic and social growth in estuarine areas, while protecting their natural features and ecological services, has become a significant priority in recent years [11]. The main objectives of this research are i) to briefly summarize the present status and recent scenario of all CZ-related issues, challenges, and management initiatives ii) to analyze the future trend of SCZM in Bangladesh.

## 2 Methodology

### 2.1 Systematic search approach and review process

**Figure 1:**
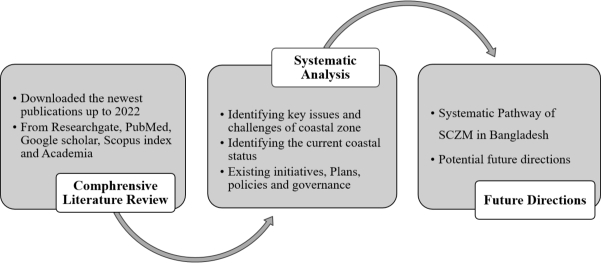
The process flow of the methodology employed at a glance.

PubMed, Research Gate, Academia, Scopus Index, and Google Scholar were used to conduct a search for the existing literature, and the results of that search, along with any others that were related, were considered as well. The following information on coastal resources utilizations in Bangladesh, present key CZ issues, previous initiatives, existing coastal zone management programs, plans, policy, and governance, as well as future recommendations for SCZM in Bangladesh have been collected from the relevant literature. Climate change, tropical cyclones, marine and coastal pollution, salinity intrusions, mangrove degradation, coastal erosion, and other current coastal issues and challenges have also been collected from the literature and discussed here. For the purpose of carrying out this review, 130 different research articles were searched and acquired. See Fig. 1.

**Figure 2:**
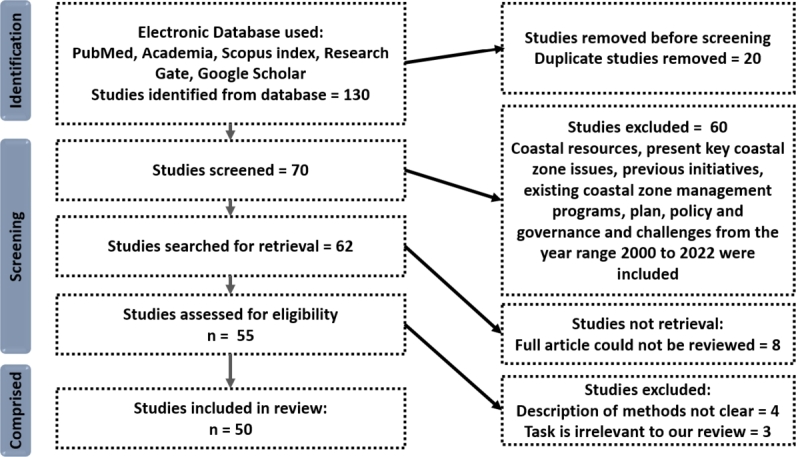
PRISMA flow diagram.

A total of 130 items were found using the database search approach. After removing duplicates, the titles and abstracts of 70 articles were reviewed. A full-text analysis was performed on 62 of these articles. After applying the inclusion and exclusion criteria, 55 papers were deemed qualified for inclusion in the review. From a coastal zone management viewpoint, the 50 papers that were chosen were carefully read and analyzed to offer a thorough analysis of the present key CZ issues, initiatives, plans, policies, governances, and difficulties associated with Bangladesh's coastal region. The PRISMA (Preferred Reporting Items for Systematic Reviews and Meta-Analyses) flow diagram, which illustrates the study selection process, provides a visual representation of the review process in Fig. 2. The publication years of the papers that were selected ranged from January 2000 all the way up to October 2022.

## 3 Coastal zone of Bangladesh

**Figure 3:**
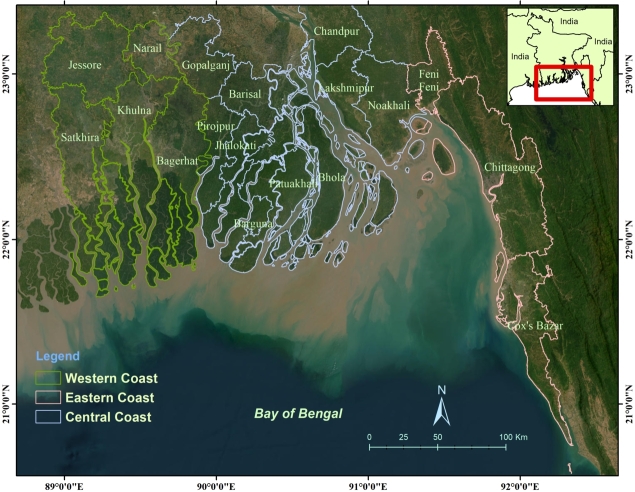
Coastal zone of Bangladesh.

The CZ of Bangladesh is located in the southern part of the country and is blessed with the largest complex estuarine systems, mangroves, coral reefs, deltas, wetlands, saltmarshes, seaweeds, coastal & marine fisheries; floras; faunas, sea salts, beach minerals, sand dunes, and other resources [5] [12] [13]. It is dominated by the Ganges Brahmaputra Meghna river system and the Bay of Bengal (BoB) from both geomorphologic ally and hydrologically. It covers an area of 47,201 km^2^ which is 32% of the total country's area, & consists of 19 districts. The CZ of Bangladesh is divided into three regions (Fig. 3) because of its different geological characteristics [14]. Hilly areas cover the eastern regions, which are more stable [15]. The central zone is characterized by the most active and continuous processes of accretion and erosion. The Meghna estuary is also located here. The western zone consists of Khulna, Satkhira, and Bagerhat districts. This region is part of the Ganges delta bordering the BoB, comprising the semi-active delta with crisscrossed by numerous rivers and channels [16]. At the southern fringe lies the Sundarbans, which is the largest mangrove forest and the source of livelihood for about 3 million people [17] [18].

## 4 Utilization of coastal resources in Bangladesh

A large portion of the population of Bangladesh benefits from different livelihood opportunities due to the use of various coastal natural resources, which also contribute to the socioeconomic advancement of the country. Participation in these activities not only causes a large number of user conflicts, but it also causes significant damage to coastal environments [9]. Because of this, it is imperative that SCZM approaches be undertaken. Bangladesh has lots of coastal and marine living, non-living resources, and renewable resources [19] (Table 1) due to its warm tropical climate, adequate annual rainfall, and vast coastal waters, which support a diverse amount of marine life [20].

**Table 1:** Resource categories of Bangladesh coastal and marine areas according to [21].

Resources Types	Categories
Living	Fisheries, Mangroves, Coral reefs, Seaweed, Seagrass, Plankton
Non-living	Minerals, Petroleum (oil & gas), Fresh water, Sea salt
Renewable	Wind, Wave, Tide, Ocean currents, Solar

### 4.1 Fisheries

Within the context of the economy of Bangladesh, the sector of fisheries plays a key role in the provision of national food security, the facilitation of individual empowerment, and the generation of foreign exchange profits [22][12]. Bangladesh is thought to be one of the best places in the world for fishing. It has the largest flooded wetland in the world and the third most aquatic species in Asia after China and India. Bangladesh has about 160 million people, and about 12% of them work in the fishing sector [23], either full-time or part-time. Fish supplies around 60% of the total animal protein that humans consume [24]. The fishing industry makes up 3.50% of the Gross Domestic Product (GDP) of the country and 25.72% of the agricultural GDP [25]. Bangladesh has received vast marine water resources from Myanmar (1,11,631 square kilometers in 2012) and India (19,467 square kilometers in 2014) as a result, the blue economy has recently attracted remarkable attention [26]. Despite the extensive coastline and vast marine water area, the marine fishing sector is still in its infancy. However, the effective usage and conservation of marine resources can enhance food security and improve lifestyles [27] without affecting the coastal ecosystems and the achievement of Sustainable Development Goal (SDG) 14 on the United Nations' agenda [28].

### 4.2 Mangroves

The largest mangrove forest in the world is Sundarbans. It is one of nature's wonders and is situated in Bangladesh's coastline region [29]. In the Bangladesh West Bengal and southeast peripheral regions of Sundarbans, millions of people, the majority of whom are fishermen, woodcutters, honey collectors, and larvae catchers, depend directly or indirectly on these forest and wetland resources for their health, food security, livelihood, and economic well-being [30]. In a study on Sundarbans ecosystems-dependent livelihood, it was found that approximately 50% of coastal households depend directly on forest resources for their livelihood support to varying degrees, e.g., extracting primarily fuel wood (74%), fish (78%), and Golpata (27%), with an estimated annual value of approximately one thousand dollars per household [31] [32].

### 4.3 Coral reefs

Saint Martin's Island is the only coral island in Bangladesh [33]. The local economy receives 33.6 million USD annually from fishing, tourism, coastal protection, seaweed cultivation, and collecting intertidal shellfish due largely to St. Martin's Island's coral reef and its associated habitats [34]. The primary contributors to the economy are the fishing and tourism industries, which bring in 19.4 and 13 million US dollars annually, respectively, in terms of direct use values [34]. Over the course of the last few decades, the island has developed into one of the most well-known tourist sites in the nation [35]. The characteristics such as the natural beauty, high quality of service, opportunities for adventure, and opportunities for leisure are what lure tourists to the island as opposed to other destinations [36].

### 4.4 Seaweeds

Seaweeds are available all over the Bangladeshi coastline, primarily in the Sundarbans Mangrove Forest, Cox's Bazar, and St. Martin's Island [37] [38]. There are 19 commercially significant species among the 193 total seaweed species that are found, which are divided into 94 genera. There are about 5,000 metric tons of seaweed biomass available. Seaweeds are typically available from October to April due to seasonal variations in water quality characteristics, although their greatest abundance is from January to March [39]. Various value-added food, functional food, and personal care products have been created by government institutions, non-profit organizations, and the business sector [39] [38].

### 4.5 Minerals

It has been determined that Bangladesh's coastal islands and coastal belt contain sand deposits that could potentially be used for beaches [40]. The following is a list of some heavy minerals: zircon, rutile, ilmenite, leucoxene, kyanite, garnet, magnetite, and monazite [41]. There are likely undiscovered mineral deposits inside the maritime bounds of the BoB; however, excessive sedimentation may make it substantially more difficult to investigate these resources, particularly in coastal regions [42]. If these resources are properly managed, this natural capital might be transformed into employment opportunities, improvements to infrastructure and public services, and expansion of the domestic private sector [26].

### 4.6 Petroleum (oil and gas)

The International Court of Justice arbitrated maritime boundary disputes with Myanmar and India in 2012 and 2014, establishing Bangladesh's sovereignty over a 1,18,813 square kilometer territorial sea [43]. As a result, the enormous potential of the blue revolution has emerged following the conquest of a new sea that is approximately the same size as Bangladesh [44]. The ocean contributes more than $6 billion to the Bangladesh economy each year and has the potential to generate even more as the oceans contain vast reservoirs of valuable commodities [45]. The maritime boundary obtained from Myanmar and India consists of 26 gas blocks [46]. According to analysts, leasing these blocks might provide access to around 40 trillion cubic feet of gas [26].

### 4.7 Sea salt production

The coast of Cox's Bazar has historically been the location in Bangladesh where the majority of the country's marine salt is produced [26] [47]. During the dry season, the areas that have the ability to produce crude salt in salt pans include onshore regions such as Chakaria and Cox's Bazar as well as offshore islands such as Moheshkhali and Kutubdia (December–May). The typical yield of such crude salt is somewhere in the range of 7,000 to 10,000 kg per hectare [48]. According to Alam (2014) [26], there are locations along the coast where certain farmers may be able to produce the salt of approximately 20,000 kg/ha/season.

### 4.8 Renewable energy

There is a massive amount of untapped potential for the production of renewable energy in the ocean, particularly in the forms of wind, wave, and tidal power, as well as biomass, thermal conversion, and salinity gradients [49]. The offshore wind energy business is currently the most developed of the ocean-based energy sources that are available [50]. The global installed capacity was just slightly more than 6 GW in 2012; however, this is expected to triple by the year 2014, and projections that are somewhat conservative imply that this might expand to 175 GW by 2035 [26] [51].

## 5 Key coastal zone issues of Bangladesh

The coastal area is known to have a lot of human involvement [52]. The coastal regions of Bangladesh show a remarkable resemblance to those places that are subject to such rigorous uses. At the present time, these regions are utilized for farming, the raising of cattle, fishing, the cultivation of shrimp, and the manufacture of salt [53]. Coastal regions are home to a variety of economic sectors, including tourism, airports, seaports, land ports, and export processing zones [26]. The CZ of Bangladesh has experienced massive urbanization and economic growth in recent years, which has created a bunch of new issues and to some extent accelerated the degradation of the coastal ecosystems and habitats [54].

### 5.1 Climate change

Bangladesh's vulnerability to the effects of climate change, particularly in the coastal region, is characterized by the country's flat and low-lying land, impoverished coastal community, and reliance of many people's livelihoods on climate-sensitive industries such as agriculture and fisheries, as well as an insufficient institutional framework [55]. The geographic location of Bangladesh makes it one of the countries that are vulnerable due to the effects of climate change and sea level rise, although it has a lower contribution to global greenhouse gas emissions, which is less than 0.1% [56]. Because of climate change, Bangladesh has been dealing with a variety of socioeconomic, environmental, and demographic challenges, such as increased inundation, vulnerability to cyclone and coastal flooding, increased drought, saltwater intrusion, and outbreaks associated with greater extremes of temperature [55]. If sea levels rise by 1 m by 2050, approximately 18% of Bangladesh's land area will be submerged, affecting coastal resources and ecosystems [57] [58].

### 5.2 Tropical cyclones

Bangladesh is considered a disaster-prone region due to being frequently affected by seasonal tropical cyclones, storm surges, tidal surges, and coastal erosion [59] [54]. It has been reported that cyclone effects cause approximately 49% of deaths on the Bangladesh coast [60], and Bangladesh is ranked as the fifth most disaster-prone country in the world [61] [59].

### 5.3 Coastal and marine pollution

The primary contributors to pollution include land runoff and sewage, as well as the mariculture and other forms of marine production and activity that take place in coastal seas [62]. Riverine exportation of agrochemicals from coastal catchments, household waste materials, and industrial waste are the most common types of pollutants that come from land-based sources [63]. The coastal area of Bangladesh is contaminated by various anthropogenic activities like agricultural, domestic, industrial wastages, sewages, farming, ship building and ship braking industries wastes are directly discharging into the water [64].

### 5.4 Coastal erosion

Coastal regions are significant from an environmental, social, and economics perspective. Because of the high population density and the many different types of development activities, such as increasing transportation and tourism activities, coastal erosion is now seen as a major problem. These activities include manufacturing and other types of industrial activity [65] [66] [67] [68]. In coastal regions all around the world, there has been rapid development reported. Bangladesh frequently experiences coastal erosion. The important coastal areas that are constantly being eroded are the Meghna estuary, the east coast of Hatiya, and the south western shore of Sandwip [69]. In the past few decades, 40% of the land on Sandwip Island has been eroded, and around 10% of the land on Kutubdia Island has been lost to erosion [70]. In the future, Bangladesh will be subjected to the serious impacts of climate change, such as sea level rise. This will amplify the impact of coastal erosion across all coastal districts, which will have a negative impact not only on the environment but also on the local economy [71] [72] [73].

### 5.5 Saltwater intrusion

Because of climate change, the coastal region of Bangladesh is seeing an increase in salinity, which is projected to have severe impacts on the supply of fresh water and brackish water for purposes such as agricultural production, drinkable water, and freshwater habitats [74]. Saline water intrusion is very significant in our country due to its exposure to the sea. The intrusion of seawater has been called one of the biggest environmental concerns because it makes it hard to grow crops along the coast of the southern part of Bangladesh [75]. The fact that salinization is happening at an unusually high level around the world shows that salts are building up, which is dissolvable in freshwaters and soil properties [76]. According to the Soil Resources Development Institute (SRDI) 2010, the saline water effect is mainly on the coastal area, which was 83.3 million hectares in 1973, 102 million hectares in 2000, and 105.6 million hectares in 2009, and it is increasing.

### 5.6 Mangrove's degradation

Due to natural extreme events like tropical cyclones, salt intrusions, sea level rise [77], overexploitation by humans, increasing multiple pressures, industrial effluents, oil spillage, agricultural runoff, and anthropogenic pollution, these, directly and indirectly, affect the biodiversity and ecosystems of the Sundarbans [78] [79] [80]. Between 1881 and 2001, the frequency of cyclones on the BoB along the Sundarbans rose by 26% [81]. Storm intensity is also predicted to increase during the pre-monsoon months of May and June, with potential effects on mangrove structure and functions [82] [83]. In Sundarban Mangrove forest sea surface temperatures have risen at a rate of about 0.05 °C each year, which is significantly greater than the warming trends seen in the tropical Pacific Ocean (0.01-0.015 °C/year) and tropical Atlantic Ocean (0.01-0.02 °C/year) [84]. The level of freshwater flow to the Sundarbans has been drastically reduced as a result of anthropogenic factors, particularly some developmental infrastructures, such as the construction of dams and extensive shrimp farming upstream, which has facilitated the salinity intrusion caused by climate change [85]. Man-made factors contributing to Sundarban's degradation include the diversion of freshwater from the Ganges at Farakka, navigation, oil pollution, the construction of coal-fired power plants, industrial pollutants, heavy metals, shrimp cultivation, pesticides, poaching, and the failure to enforce laws. Pollutants accumulated during dry periods are washed away and carried to the BoB during the monsoon. It is expected that the accumulated pollutants will have a long-term impact [86].

### 5.7 Others issues

Other issues include unplanned tourism, multiple stakeholders, overexploitation of coastal and marine resources, unplanned coastal structural development, seashell and coral extractions, habitat destruction, human accessibility, unsustainable management plans, and so on [87].

## 6 Existing coastal zone management program in Bangladesh

Several initiatives for the conceptualization of integrated coastal zone management (ICZM) in Bangladesh have been taken by the government of Bangladesh (GoB) [88]. In Bangladesh, the ICZM process was simplified by a program development office that was set up in 2001 under the 1999 policy directives. They are initiatives [7] [89].

• Off-Shore Islands Development Board (1977–1982)
• Coastal Environment Management Plan for Bangladesh (1987)
• Special Parliamentary Committee on Coastal Area Development (1988–1990)
• Coastal Area Resources Development Plan (CARDMA) (1988)
• National Tourism Policy (1992)
• National capacity building approach the ICZM initiative (1997)
• Tsunami vulnerability map (2005)
• Coastal Zone Policy (CZP) (2005)
• Coastal development strategy (CDS) (2006)
• The National Tourism Policy (2009)
• Bangladesh Delta Plan (BDP) 2100

All stakeholders, including concerned ministries, local and regional agencies, governmental organizations, non-governmental organizations, and civil society, should put their efforts into following the ICZM principles for the development of the CZs of Bangladesh.

**Coastal Zone Policy (CZP), 2005** To achieve and implement the overall goals of the ICZM coastal zone policy of 2005, a very important action plan is needed in Bangladesh. The 2005 formulation of CZP by the GoB provides an overall policy framework or guidelines for the management and development implementation in the CZ of Bangladesh [3]. The general guidelines regarding CZP for 2005 are: i) Economic growth ii) Basic needs and opportunities for livelihoods iii) Reduction of vulnerabilities iv) Sustainable management of natural resources v) Equitable distribution vi) Empowerment of communities vii) Women's development and gender equity viii) Conservation and enhancement of critical ecosystems.

**Coastal Development Strategy (CDS), 2006** CDS based on the CZP 2005 is a targeted process and doesn't represent overall development. The targeted objects are regions, disadvantaged groups, issues, and opportunities. Nine strategic priorities are conservation, women's empowerment, improving livelihood conditions, optimizing use, and sustainable resource management [90].

**ICZM Program (2006-2010)** For the implementations of CZP 2005, the program is regarded by the government (duration: January 2006–December 2010) as an effort to establish ICZM in Bangladesh under consideration of the CDS and CZP and the institutionalization and operationalization of ICZM approaches in Bangladesh [3].

### 6.1 Community-based coastal zone management in Bangladesh

#### 6.1.1 Cyclone preparedness program (CPP)

**Figure 4:**
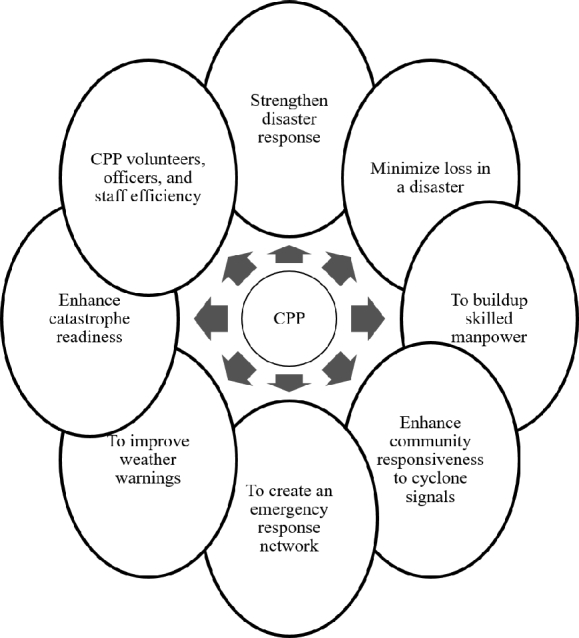
Objectives of CPP in Bangladesh [94].

The CPP of the GoB and the Bangladesh Red Crescent Society was founded in 1972 after the cyclone of 1970 [91]. The purpose was to notify the coastal communities about cyclone forecast, warning, first aid, rescue, and relief [92] [93]. The CPP is a one-of-a-kind institutional system for community preparedness that was developed to lessen the impact of the devastation caused by the catastrophic tropical cyclone that regularly strikes the coast of Bangladesh. The objectives of CPP are given in Fig. 4.

#### 6.1.2 Cyclone shelter centers

The funnel-shaped northern region of the BoB magnifies the storm surge of landfilling tropical cyclones, making Bangladesh vulnerable to the effects of cyclones [95]. The deadliest cyclone in recorded history struck in 1970, causing widespread destruction and more than 300,000 deaths [96]. Cyclone Sidr, which struck in 2007, was responsible for the deaths of 3,500 people; Cyclone Fani, which struck in 2018, took the lives of fewer than 10, and Cyclone Amphan, which struck in 2020, killed approximately 20 people. The government forecasts that in order to strengthen disaster resilience in a total of 19 coastal areas by the year 2025, they will require more than 7,000 shelters that may serve several purposes [97]. Along the length of Bangladesh's coastline, which is around 710 kilometers long, there are approximately 2,500 cyclone shelters and multifunctional cyclone shelters. Since the disastrous cyclone that occurred in 1970, they have been gradually developed in order to give a safe haven facility for the people who live along the coast. The provision of cyclone shelters is the primary measure that the government has taken to implement cyclone catastrophe mitigation [96].

#### 6.1.3 Char development and settlement project (CDSP)

In order to enhance the economic condition of the people who now live in the coastal chars, the GoB plans to introduce productive human settlements into the coastal chars. The Land Reclamation Project (LRP) was initiated by the government in order to minimize the social, structural, and environmental vulnerability that was present in char areas. The LRP was carried out by the Bangladesh Water Development Board (BWDB) between the years 1979 and 1991 with financial assistance from the government of the Netherlands. This project was eventually succeeded by the CDSP, also known as the CDSP. With BWDB treated as the project's lead agency, the CDSP evolved into a multi-agency integrated project that included the participation of the Ministry of Land, the Local Government Engineering Department, the Department of Agricultural Extension, the Department of Forestry, and the Department of Public Health Engineering [98].

#### 6.1.4 Coastal green belt

A green belt is a section of land that has been preserved as open space, and it is typically located in the suburbs of larger cities [99]. In the twenty-first century, the idea of a “green belt” plays a significant role in the process of environmentally responsible growth [100]. It is necessary to have a greenbelt in order to defend the embankment from tidal surges using plantings on the outer slope of the embankment, as well as to protect life and property in the region using plantings on the embankment [99]. In Bangladesh, a coastal afforestation program was started in 1965. According to the Department of Environment [101], trees covered 93% of the mangrove plantations planted by the Bangladesh Forest Department in 2013 on approximately 209,140 hectares of Bangladesh's coastal area in 2015.

#### 6.1.5 Flood embankment

Bangladesh is well known for having a significant risk of floods due to its deltaic physical environment and dense population [78] [102]. Multiple flood threats, including storm surge-induced floods, fluvial-tidal floods, and pluvial floods—inundations brought on by monsoon precipitation are present in the coastal region [103] [104] [59] [105] [106]. In order to safeguard communities and agricultural land from the effects of many disastrous floods, the Bangladeshi government built coastal embankments in the 1960s [107] [108] [109]. The development of embankments along flood-prone areas protects against coastal damages, erosions, environmental losses, and ecosystem losses. The total land coverage of flood embankments now in Bangladesh is 5.37 million ha, which is about 37% of the total country area and 56% of the total cultivable lands [109].

### 6.2 Ecosystem based coastal zone management in Bangladesh

#### 6.2.1 Marine protected area (MPA)

Bangladesh has declared “Swatch-of-No-Ground” its first MPA for the protection and conservation of marine mammals like dolphins, porpoises, sharks, whales, etc. [110], which is about 1.46% of the total maritime area [110]. But it is needed to declare at least 10% of the marine area as a protected area to reach the Aichi target 11. Bangladesh declared Nijhum Dwip as a MPA in 2019 under the provisions of clause 28 of the 1983 marine fisheries ordinance. It is covered by 3188 km^2^ of estuarine waters to protect marine biodiversity and sustainable fishing activities and livelihoods [111]. In accordance with articles 13(1) and 13(2) of the Wildlife Act-2012, the ministry designated a maritime area measuring 1,743 square kilometers as the “St. Martin's Marine Protected Area” in 2022. The island's 590 hectares had already been designated as an ecologically critical area. To preserve its unique biodiversity and habitat, the government designated St. Martin's island as a MPA [112].

#### 6.2.2 Marine reserve

Bangladesh has declared 698 km^2^ of water as a marine reserve in the middle ground and south patch areas of the BoB, according to the marine fisheries ordinance of 1983 [113].

#### 6.2.3 Wildlife sanctuary

The Bangladesh government has declared many protected areas, among which a few are related to coastal, riverine, and estuarine waters for the protection of biodiversity. According to the Forest Department's 2021 forecast [114], they are Table 2.

**Table 2:** Wildlife sanctuaries in Bangladesh [114].

Wildlife Sanctuaries	Location
Nazirganj Dolphin Sanctuary (2013)	Pabna
Shilanda-Nagdemra Dolphin Sanctuary (2013)	Pabna
Nagarbari-Mohanganj Dolphin Sanctuary (2013)	Pabna
Sundarban (East) Wildlife Sanctuary (2017)	Bagerhat
Sundarban (West) Wildlife Sanctuary (2017)	Satkhira
Sundarban (South) Wildlife Sanctuary (2017)	Khulna
Pankhali Dolphin Sanctuary (2020)	Khulna
Shibsha Dolphin Sanctuary (2020)	Khulna
Vadra Dolphin Sanctuary (2020)	Khulna
Char Kukri-Mukri Wildlife Sanctuary (1981)	Bhola
Teknaf Wildlife Sanctuary (2009)	Cox's Bazar

#### 6.2.4 Ecologically critical area (ECA) in Bangladesh

It is defined as the areas where ecosystems are adversely affected by human activities. ECA to safeguard the marine environment in terms of biodiversity, crucial habitats, marine organisms breeding, protection, and mangrove reconstruction [101] Table 3.

**Table 3:** Coastal and marine ECA in Bangladesh [101].

ECA	Location
Cox's Bazar-Teknaf Peninsula (1999)	Cox's Bazar
Sundarbans (10 km landward periphery) (1999)	Bagerhat, Khulna, Barguna, Pirojpur and Satkhira
St. Martin's Island	Teknaf upazila, Cox's Bazar
Sonadia Island	Moheshkhali, Cox's Bazar

### 6.3 Delta plan 2100

**Figure 5:**
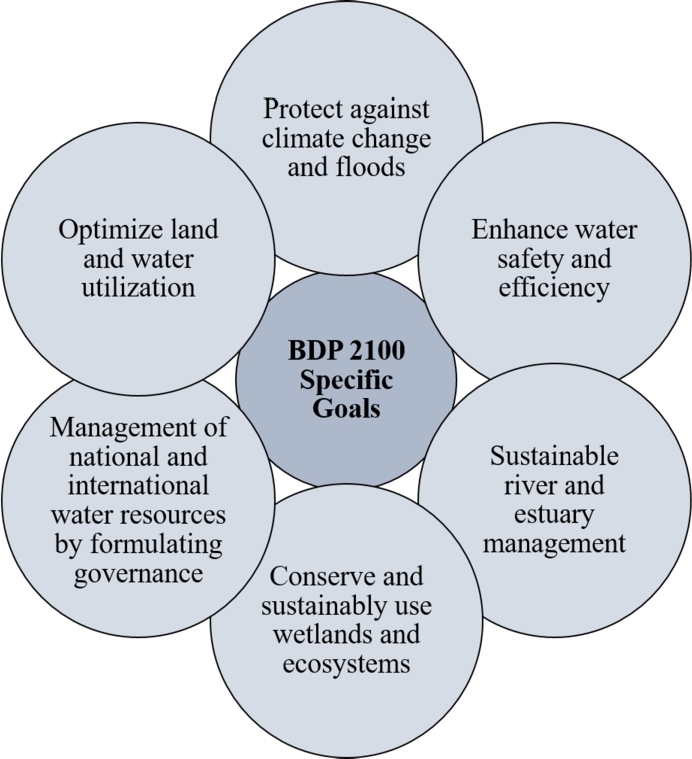
BDP 2100 specific goals [116].

The Bangladesh Delta Plan (BDP) 2100 is a long-term, integrated techno-economic mega plan that incorporates all sector programs and strategies related to the delta. It includes a Delta Vision and objectives (Fig. 5) that enable the integration of sector plans and policies over the long term and the presentation of practical interventions with a timeline for implementation. The GoB formulated a plan called “Delta Plan 2100” in 2018 under consideration of the long-term challenges of climate change and natural hazards in Bangladesh, which will take Bangladesh way forward in the next 100 years with the joint collaboration of the Government of the Netherlands [115]. It is a holistic and integrated approach to achieving sustainable development in our country, which will provide us with the complete strategy for the development of our delta [88].

### 6.4 Ocean governance framework

The Planning Commission of Bangladesh proposed a plan for the 7th Five-Year Plan (2016-2020), an integrated ocean governance framework for the management of the environment, regional collaboration, island, and river management, and the conservation of ecosystems [117].

## 7 Sustainable coastal zone management way forward

**Figure 6:**
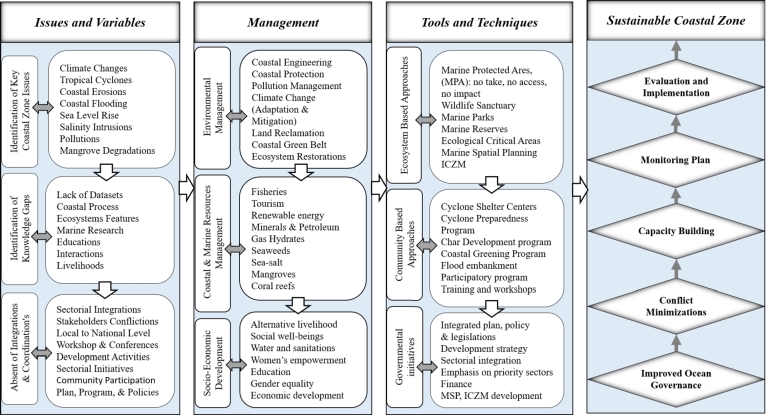
Systematic pathway of SCZM in Bangladesh.

The goal of achieving SCZM in Bangladesh requires a systematic pathway that involves several step-by-step procedures, as explained in Fig. 6. These procedures provide a framework for addressing the challenges and shortcomings that hinder sustainable management. These are issues and variables-management tools and techniques-sustainable coastal zone.

• Issues and Variables: This step involves identifying and understanding the various issues and variables that are relevant to coastal zone management in Bangladesh. These could include key CZ issues such as coastal erosion, sea-level rise, pollution, habitat degradation, population growth, and socioeconomic factors. Identification of knowledge gaps and the absence of integration and coordination are also included.
• Management: Once the issues and variables have been identified, the next step is to develop management strategies to address them. This involves socio-economic development, environmental management, and coastal and marine resources management.
• Tools and Techniques: To implement management strategies effectively, various tools and techniques are required. These tools can include both technical and policy-oriented approaches. Ecosystem-based management, which emphasizes preserving and restoring ecosystems and their services, is an important tool in SCZM. Additionally, community-based management involves engaging local communities in decision-making processes and empowering them to participate actively in coastal resource management. Governmental initiatives and policies also play an essential role in providing a supportive framework for sustainable management.
• Sustainable Coastal Zone: Improved ocean governance will minimize conflict among the stakeholders and build capacity among them. Finally, the monitoring plan, like evaluation and implementation, will lead to SCZM in Bangladesh in the near future.

More details of the future directions were explained in the following.

### 7.1 Identification of knowledge gap

**Figure 7:**
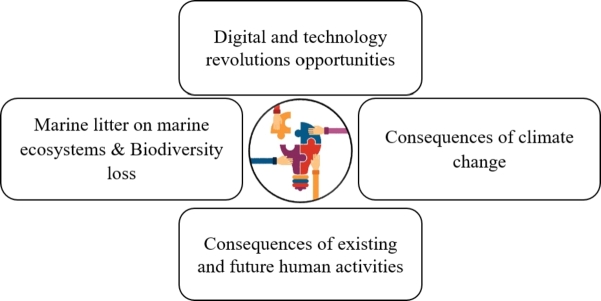
Key knowledge gaps in sustainable coastal management [119][120].

The coastal population of Bangladesh lacks sufficient knowledge of the environment and oceans Fig. 7. The majority of them lack formal education and rely on fishing and farming for their income. These individuals should be educated by the government so they may learn about environmental and ocean issues and stop being uneducated and malnourished [118]. Lack of information and data about coastal and marine resources, coastal physical and biological processes, and ecosystem characteristics prevents the effective implementation of SCZM.

### 7.2 Sustainable resource management

#### 7.2.1 Sustainable fisheries management

Marine and coastal fisheries are under threat due to overfishing, illegal, unregulated, unreported, and unwanted hunting, as well as anthropogenic activities. By reducing these unregulated fishing activities, it is able to maximize the benefits, avoid deleterious changes to stocks and the environment, and achieve stability in the fishing sector [121]. The ecosystem approach to fisheries (EAF) intends to create sustainable fisheries by connecting the broad ecological sustainability of stocks with the socio-economic viability of the fishing sector at regional and local scales [122]. This method is also known as the ecosystem-based management approach. This strategy is designed to ensure that marine food webs are in a sustainable state (i.e. there is no overexploitation), which will allow fishing to continue while also supporting human well-being. Recent studies have highlighted the significance of applying EAF to examine fishery management scenarios. This is in contrast to the existing state of fisheries management, which focuses mostly on assessment models for individual species [122] [123] [124].

#### 7.2.2 Sustainable tourism management

Unplanned tourism activities make the coastal areas more polluted and threaten the ecosystems and biodiversity. In order to mitigate the system of coastal community deterioration, a sustainable strategy is required that takes into account the physical environment, culture, and local economy. This strategy also needs to include local industries that are unrelated to coastal tourism [125] [126] [127] [128] [129]. When formulating a tourism development plan, there needs to be a comprehensive policy for sustainable development that includes decision and policy-makers, planners and managers from GOs, NGOs, and other organizations, the private sector, and the local and indigenous populations. In order to ensure the local community's well-being and a just distribution of resources and profits, including cross-cultural sensitivity, there must be a balance between conservation and people. This means meeting their demands for simple access to essential services and the capacity to manage their own natural resources [126] [127] [128] [129] [130].

#### 7.2.3 Emphasis on renewable source of energy

The requirement for the advancement of marine blue energy has been highlighted by the growing need to replace fossil fuels with alternative energy sources free from the threat of ozone depletion, with lower environmental costs and ecological footprints [131]. As non-renewable energy resources are decreasing day by day, so need to put more emphasis on renewable sources of energy, as this energy cannot be exhausted and is constantly renewed.

### 7.3 Sustainable environmental management

#### 7.3.1 Coastal engineering/protection

Beach and coastal erosion, shoreline erosion, dune erosion, impacts on coasts from severe storms, floods, increasing sea levels, and loss of biodiversity and habitat are some of the biggest problems that coastal regions are now experiencing. For the protection of the coastal environment, it needs to be assessed, evaluated, analyzed, and intended to protect against challenges like storms, waves, tides, and currents, and how they affect different ecosystems and infrastructure. Coastal engineering or coastal protection for the reduction of harmful coastal wave actions, erosions, and other natural hazards. Although some actions are being taken in some coastal areas of Bangladesh, such as the installation of geo-bags, sandbags, and retaining walls in Saint Martin's Island and Cox's Bazaar, they are insufficient [132].

#### 7.3.2 Climate change adaptation and mitigation

Climate change adaptation and mitigation are critical for Bangladesh to reduce the effects of climate change. Adaptation is an important strategy to reduce the effects of climate change. Changing planting times, adopting new agricultural varieties, cultivating crops on a homestead, planting trees, and migrating is a few of the essential adaptation strategies [133]. Even if certain nations and coastal areas are prepared with the adaptive capacity to lessen the effects of climate change, others have fewer options available to them. Because of differences in geography and economy, some coastal communities are more vulnerable than others and may have less access to food, water, and other resources than anybody else [134]. On the other hand, community participation can also help to mitigate the effects of climate change. Access to the right technology, institutions, and regulations, as well as awareness about and perspectives of climate change, all have an impact on an individual's ability to adapt [135] [136] [137] [138] [139] [140].

#### 7.3.3 Coastal and marine pollution management

Different governmental bodies in Bangladesh should come forward and take initiatives to monitor all sources of marine pollution, including land-based activities, shipbreaking, shipbuilding, tourism, oil, heavy metals, plastics, and so on, for the long-term management of marine and coastal pollution. Bangladesh requires a significant number of ocean affairs bodies to deal with the many people in the community working to safeguard the marine environment [141]. Coastal and marine pollution makes coastal ecosystems threatened. Identifying the sources of pollution, making an effluent treatment plant, and applying 3R (reuse, reduction, and recycling) are necessary for coastal and marine pollution management.

#### 7.3.4 Coastal land reclamation

It is the process of forming new land from seawater. Land reclamation minimizes the land-user conflict, solves the need for land resources, develops the economy, and provides environmental protection [142].

#### 7.3.5 Ecosystem based approaches

The ecosystem-based management approach, also known as environmental management, takes into account the interactions of species within an ecosystem, including humans [143]. The main goal is to maintain healthy, productive, and resilient ecosystem conditions so that they can provide human goods and services [144].

#### 7.3.6 Ensuring communities' participation

Participation from the local community is becoming an increasingly popular idea as a means to improve the sustainability of development efforts. Community participation is needed for SCZM. By ensuring community participation, it is possible to reduce post-disaster vulnerabilities [145]. Therefore, when utilizing community-based natural resource management in post-disaster rehabilitation, external organizations are required to work as coordinators to establish partnerships with local citizens in order to conserve natural resources, maintain livelihoods, and safeguard healthy ecosystems [146]. Global development organizations, which provide the majority of the funding for disaster preparedness programs, are becoming more aware of the importance of community engagement, education, and awareness campaigns to reduce risk in coastal areas. Local NGOs are also actively addressing these issues locally.

#### 7.3.7 Socioeconomic development

Socioeconomic development is the key element for ICZM. ICZM is very important to reach the SDGs in our country.

### 7.4 Building integration and coordination

#### 7.4.1 Strengthening stakeholder's engagement

It is crucial to develop well-managed engagement processes for building strong local stakeholder involvement. These processes should take into consideration the cultural, scientific, sociological, economic, and political settings that are the foundation of effective stakeholder participation [147]. The coastal zone management process is not an isolated process, so it is important to build integration and coordination among different stakeholders in the coastal regions as well as integrate it with our national development.

#### 7.4.2 Integrated plan, policy and law

A policy plan based on sectors will not help to achieve SCZM. This requires a national not a regional, integrated plan, policies, and laws. As a result, governmental laws and policies, as well as their strict enforcement, must be integrated. If a comprehensive maritime policy is not put into place, it will not be feasible to accomplish the objective of sustainable development through the expansion of the blue economy [52].

#### 7.4.3 Ensuring political support

Political backing contributes to the success of the ICZM process. Representation of different regional and local integrated programs is required to ensure political support [7].

#### 7.4.4 Conflict minimization and stakeholders engagement

The government is responsible for identifying all of the key stakeholders and organizing seminars and workshops to bring those stakeholders together so that they may communicate with one another. Political and administrative stakeholders in Bangladesh are active participants in the implementation of each issue. Non-public stakeholders are not involved in the policy-level discussions. It is important for the government to make an effort to involve these nonpublic stakeholders in the policymaking process [52]. Conflict minimization among different sectors and stakeholders is an essential element of a sustainable coastal management process. So, need integration in planning, which will minimize the conflict.

#### 7.4.5 Emphasis on priority sectors

It is possible to maximize resource utilization by focusing on priority sectors and special groups such as women and children, fishermen, island communities, special regions, renewable resources, fisheries resources, and so on.

### 7.5 Development tools and techniques

#### 7.5.1 Marine spatial planning (MSP)

MSP is now developed worldwide for sustainable coastal and ocean management, which aims to organize the spatial and temporal uses of resources like fisheries, aquaculture, tourism, shipping, renewable energy, and the interactions between the user and environment [148]. It reduces conflict among resource user groups, ensures conservation, balances economic, social, and environmental goals, and promotes ecosystem-based coastal management.

#### 7.5.2 ICZM in Bangladesh

**Figure 8:**
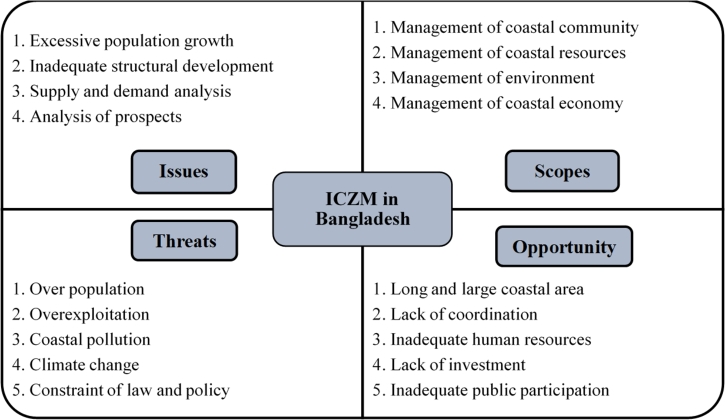
Issues, scopes, threats and opportunity of ICZM in Bangladesh [88].

**Figure 9:**
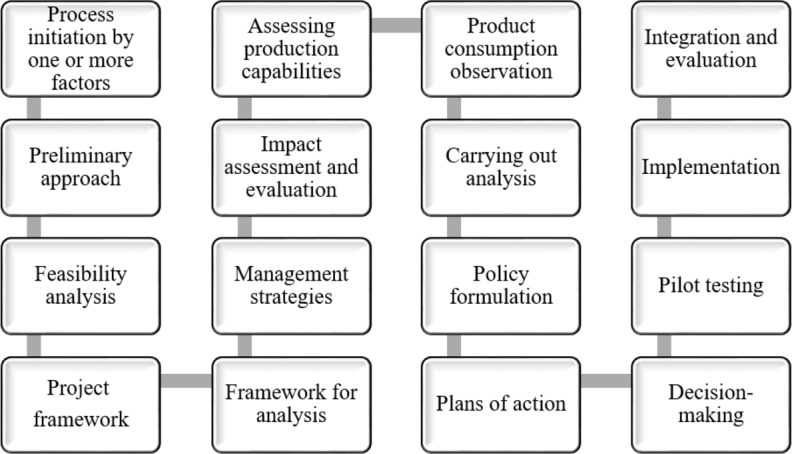
ICZM planning process ([149]).

It is the management systems of an integrative and holistic approach and an interactive decision-making process for the challenging issues in the coastal region that sustain the coastal process while maintaining integrity, reducing user conflict, maintaining environmental health, and developing multi-sectoral functions [149]. Issues, scopes, threats and opportunity of ICZM in Bangladesh explained in Fig. 8. According to Thia-Eng (1993) [149] ICZM planning process has given in Fig. 9

#### 7.5.3 Improved ocean governance

The most significant problem in ocean governance mechanisms is possibly the failure to apply current international instruments. Numerous regional tools are added to the overall framework for ocean governance, frequently in conjunction with national laws [150] [151]. Improved ocean governance to ensure transparency and accountability is one of the most important tools for SCZM. For SCZM in Bangladesh, improved ocean governance is very essential. A suitable legal framework is an essential instrument for achieving SDG 14, which will ensure the continued health of the coastal and marine environment if the associated targets are met [52].

### 7.6 Implementation and evaluation

Finally, measure the implementation to ensure that management activities are carried out. It is important to monitor the management activities at all levels, from local to national.

## 8 Conclusion

SCZM plays a crucial role in driving sustainable development in Bangladesh, particularly in attaining the country's SDGs. Target 14.1 of the SDGs aims to prevent and substantially decrease marine pollution, especially stemming from activities on land such as marine debris and nutrient pollution, by the year 2025. Meanwhile, target 14.2 strives to effectively manage and safeguard marine and coastal ecosystems in a sustainable manner, avoiding significant detrimental effects. This involves making them more resistant to damage, engaging in restoration efforts, and eventually working towards the objective of creating thriving and productive seas as a whole. There is highly required for SCZM. As the CZs of Bangladesh are facing many threats and pressures due to anthropogenic activities and natural hazards, so immensely need to manage the CZs in a sustainable way.

## CRediT authorship contribution statement

All authors listed have significantly contributed to the development and the writing of this article. **Mosa. Tania Alim Shampa**: Conceived and designed the experiments; Performed the experiments; Analyzed and interpreted the data; Contributed reagents, materials, analysis tools, or data; Wrote the paper. **Nusrat Jahan Shimu**: Performed the experiment; Analyzed and interpreted the data; Wrote the paper. **K M Azam Chowdhury**: Conceptualization; Analyzed and interpreted the data; Supervision. **Md. Monirul Islam**: Conceived and designed the experiments; Wrote the paper; Supervision. **Md. Kawser Ahmed**: Conceptualization; Supervision.

## Declaration of Competing Interest

The authors declare that they have no known competing financial interests or personal relationships that could have appeared to influence the work reported in this paper.

## Data Availability

Data will be made available on request.
